# Supplementary material for: Amelioration of Sensorineural Hearing Loss through Regulation of Trpv1, Cacna1h, and Ngf Gene Expression by a Combination of Cuscutae Semen and Rehmanniae Radix Preparata
Source: Nutrients. 2023 Apr 5;15(7):1773. doi: 10.3390/nu15071773 (PMC10097224; doi:10.3390/nu15071773)
Supplement: Supplementary file 1 [file nutrients-15-01773-s001.zip › nutrients-2278995-supplementary.pdf]

Supplementary Material

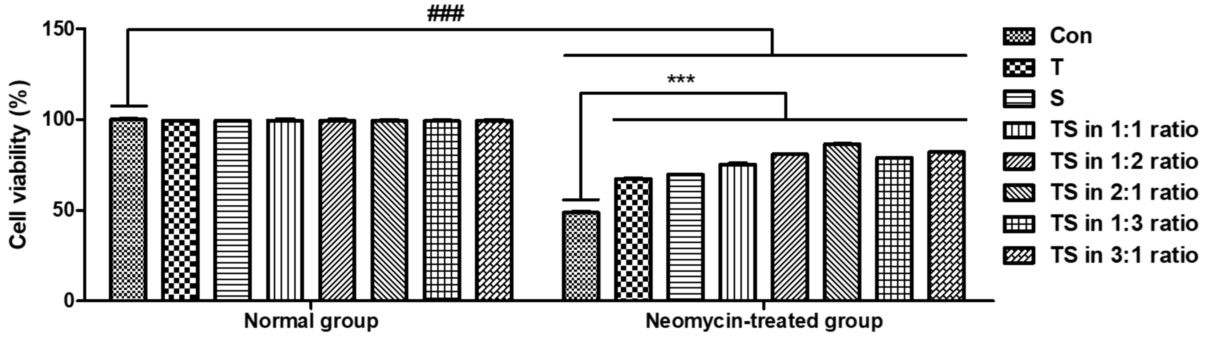

Figure S1. Ratio assessment of Cuscutae Semen (T) and Rehmanniae Radix Preparata (S) combination using MTT assay in HEI-OC1 cells. Data are presented as means ± SEM of three independent experiments in triplicates. $^{###}p < 0.001$, $^{***}p < 0.001$.
